# Service-Aware Clustering: An Energy-Efficient Model for the Internet-of-Things

**DOI:** 10.3390/s16010009

**Published:** 2015-12-23

**Authors:** Antoine Bagula, Ademola Philip Abidoye, Guy-Alain Lusilao Zodi

**Affiliations:** 1 Intelligent Systems and Advanced Telecommunication Laboratory, Department of Computer Science, University of the Western Cape, Private Bag X17, Bellville, Cape Town 7535, South Africa; 3008758@myuwc.ac.za; 2 Department of Computer Science, Namibia University of Science and Technology (NUST), Private Bag 13888, Windhoek 9000, Namibia; gzodi@nust.na

**Keywords:** service-aware clustering (SAC), Internet-of-Things (IoT), energy efficiency, clustering mechanisms, hybrid sensor networks

## Abstract

Current generation wireless sensor routing algorithms and protocols have been designed based on a myopic routing approach, where the motes are assumed to have the same sensing and communication capabilities. Myopic routing is not a natural fit for the IoT, as it may lead to energy imbalance and subsequent short-lived sensor networks, routing the sensor readings over the most service-intensive sensor nodes, while leaving the least active nodes idle. This paper revisits the issue of energy efficiency in sensor networks to propose a clustering model where sensor devices’ service delivery is mapped into an energy awareness model, used to design a clustering algorithm that finds service-aware clustering (SAC) configurations in IoT settings. The performance evaluation reveals the relative energy efficiency of the proposed SAC algorithm compared to related routing algorithms in terms of energy consumption, the sensor nodes’ life span and its traffic engineering efficiency in terms of throughput and delay. These include the well-known low energy adaptive clustering hierarchy (LEACH) and LEACH-centralized (LEACH-C) algorithms, as well as the most recent algorithms, such as DECSA and MOCRN.

## 1. Introduction

Next generation sensor networks are predicted to be deployed in the Internet-of-Things (IoT) using heterogeneous sensor fabrics and following a service architecture where the sensor nodes/motes equipped with different sensing, identification and communication capabilities are tasked to deliver different services to different users anytime, anywhere and using anything to reach some of the areas of our life that we could not fathom without the advances made in the sensor technology. However, current generation wireless sensor systems have only scarcely exploited the integration of services, such as detecting and locating an object within an environment [1] while giving an indication whether that environment is suitable for the object to be. A hybrid IoT system, which is able to provide such integration by combining sensor and radio frequency identification (RFID) technologies, can be highly useful for many industries. In the mining industry, for example, sensors can be used to prevent explosions due to methane gas seeping from the surrounding strata [2,3], while RFID could be used to provide information about whether there are miners within the vicinity of dangerous concentrations of gases [4]. Similarly, when deployed in supply chain management, a hybrid IoT model can be used when transporting fresh produce from the farm to the supermarket, by using sensor devices to verify whether the vehicle or warehouse in which the produce is stored is at the correct temperature in order to preserve the quality of the produce. Smart RFID tags equipped with temperature sensors are then simultaneously used to sense the surrounding environment’s temperature and send an alert if the produce is being exposed to too low or high temperatures. Various other smart applications derived from the integration of sensor and RFID technologies can be beneficial to different industries. These include the identification, counting and detection of human presence and the control of the heating, ventilation, air conditioning and lighting in smart buildings, patient and staff identification and tracking [5] in healthcare systems and the design of smart parking systems in smart cities. Separate deployment of the sensor and RFID technologies may lead to a duplication of both hardware and software resources, complex and costly system management and difficulties in system trouble shooting and maintenance.

Researchers [6,7,8,9] have presented different ways of integrating both technologies with the objective of building an *ad hoc* network that has similar properties to wireless sensor networks (WSNs). The issue with such an approach is that the resulting network will also present similar energy limitations to WSNs. In [6], on-demand wakeup is used to eliminate idle listening with the expectation of energy savings. While resulting in energy savings, this approach uses the medium access control (MAC) protocol, which is not a natural fit for the mesh model implemented by sensor networks. Other approaches have been proposed for the integration of sensor and RFID technologies. This includes the work done in [7], where an integrated sensor-RFID node was designed to enable sensing of gas levels in underground mines and detecting the presence of miners while providing communication capabilities using ZigBee or WiFi. The designed sensor/RFID mote was further used as a sensor prototype in the smart parking prototype described in [8] and as a sensor node in the optimal placement model for smart parking described in [9]. However, despite their relative advantages compared to separate sensor/RFID deployments, these approaches may lead to heterogeneous energy consumption patterns, which need to be accounted for when routing the sensor readings from their locations to the base station of a hybrid IoT system. Otherwise, the network may experience an energy imbalance and subsequent short-lived IoT infrastructures resulting from routing the sensor readings over the most service- intensive sensor nodes, while leaving the least active nodes idle.

### 1.1. Sensor Network Clustering

The issue of energy efficiency for wireless sensor networks has attracted much interest in the research community, as these networks are built around tiny nodes with limited processing, minimum memory footprint and low communication capabilities. Sensor network clustering is an energy-aware traffic engineering method, which aims to maximize the lifetime of a sensor network, by structuring the sensor network into clusters and assigning “cluster head” and “cluster member” roles to the sensor nodes. This enables multi-hop communication, data aggregation and fusion within a cluster, in order to decrease the number of messages routed by the sensor network and essentially reducing the number of hops traversed by the sensor readings from the source to the sink node. This is implemented by subdividing a sensor network into a hierarchy of sensor clusters, each containing a cluster head. The sensor nodes are organized into cluster members and cluster heads depending on different performance parameters, such as their residual node’s energies. The cluster heads play the role of collecting, aggregating and processing data, while the cluster members are assigned the minimal functioning roles of sensing and forwarding. By producing several points of data aggregation and processing in the core of the network, in-network processing is promoted, and the network edge is left light. This model moves away from the Internet paradigm, which uses intelligent edges to produce data, which are forwarded by a dumb core.

Low energy adaptive clustering hierarchy (LEACH) [10] and LEACH-centralized (LEACH-C) [11] are two clustering algorithms that have been proposed to address the energy efficiency issue in wireless sensor networks. While LEACH builds upon a distributed clustering model where the sensor nodes independently determine their role based on a probabilistic value or residual energy, LEACH-C uses a centralized model, where the gateway attached to the sink node uses *a priori* knowledge of the geographical position, residual energy and neighbor information of all sensor nodes to select the cluster heads. However, both LEACH and LEACH-C are built around a homogeneous service architecture, where all sensor motes are assumed to be made of the same sensor fabric and are expected to deliver equal service and exhibit the same power consumption profile for all nodes. This discounts the energy required to deliver these services, thereby leaving the energy consumption equation unsolved.

DECSA [12] is an improvement of the the LEACH protocol, which considers both the residual energy of nodes and the distance between nodes when selecting cluster heads (CHs) during cluster formation. It is based on a three-level hierarchical structure that divides a sensor network into four types of sensors: common sensor nodes (SN), cluster head node (CH), base station cluster head (BCH) and base station (BS). In DECSA, the selection of CHs depends on a random number generated by each sensor node. This random number must be less than a predefined threshold. The sensor node with the highest probability becomes a CH depending on the node’s residual energy. DECSA avoids direct communication between the CH and the base station when their communication distance is greater than a given threshold value. It reduces energy consumption by 40%, prolongs the network’s lifetime by 31% and performs better compared to the LEACH protocol. However, the cluster heads selected based on this approach are not uniformly distributed within the network, and there is a high probability that the selected CHs will be on the same side of the network. This might result in an increase in energy consumption.

MOCRN [13] is another recent improvement of the LEACH protocol, which is based on the minimum separation distance between the cluster heads. Initially, MOCRN randomly selects K nodes as its cluster heads (CHs). Every node selected as a CH sends its information to the next node within its transmission range. The neighbor nodes that receive this information do likewise and transmit it to the next neighboring nodes, and the process continues, until the node meets a neighbor node contained in another cluster. Through this process, each local cluster is formed, and the number of clusters is the same as the number of cluster heads. Thus, choosing the number of CHs is the same as choosing the clustering size. In MOCRN, communication in the sensor network is divided into intra-cluster communication (using a single hop between cluster members and their cluster head) and inter-cluster communication (using multiple hops between cluster heads). MOCRN is a protocol that uses a simple process for the formation of clusters where CHs are selected based on the minimum distance between the nodes, but discounts the residual energy of the selected nodes, which may not have enough energy to receive, aggregate and re-transmit the data to the next CH or the base station.

As described above, LEACH, LEACH-C, DECSA and MOCRN are energy-efficient protocols, which have been designed based on an energy-aware, but service-blind routing approach, where the sensor nodes/motes are assumed to have the same sensing and communication capabilities and also provide the same services. These routing algorithms are not fit for the next generation IoT, where the heterogeneity of services will lead to heterogeneous devices with different energy consumption profiles. Such a diversity of energy consumption profiles should be accounted for when designing next generation energy-efficient IoT platforms.

### 1.2. Contributions and Outline

It is widely recognized that clustering techniques may help alleviate the energy issue in sensor networks. However, the application of traditional “service-blind clustering (SBC)” algorithms to the IoT may lead to energy imbalance, arising from electing the most service-demanding nodes as cluster heads and aggregating and forwarding most of a cluster’s sensor readings to the base station, while the least service-demanding nodes are used as cluster members, which remain idle most of the time. “Service-aware clustering (SAC)” is a natural fit for the IoT platforms where the heterogeneity and diversity of service profiles of the smart objects demand new ways of engineering the multitude of interconnected sensor networks islands. However, to the best of our knowledge, traffic engineering techniques that account for such service profiles have not yet been addressed by either the research or the practitioner communities. This paper’s main contribution is to propose and assess the relevance of using a traffic engineering model that builds upon clustering techniques and sensor nodes service profiles to improve the lifetime of a wireless sensor network. The proposed TEmodel is built around four main ideas.

#### 1.2.1. Energy Awareness

We consider a model where different energy levels are associated with different roles the sensor nodes can play in a clustering process and where the sensor nodes are allocated different roles in the network based on their residual energy: energy-depleted nodes are allocated lower energy-demanding roles as edge nodes playing the role of cluster member, while fully-energized nodes are allocated high energy-demanding roles as core nodes playing the role of cluster heads.

#### 1.2.2. Service Awareness

We consider a model where the sensor motes/nodes are classified into service classes depending on their service profile and fabric and are then assigned corresponding roles. The least service-demanding nodes have a higher probability of becoming cluster heads in the core of the network tasked for collecting, aggregating and forwarding data to the base station, while the most service-intensive ones are left as member nodes at the edge of the network tasked for collecting data and forwarding to the cluster head.

#### 1.2.3. Centralized Routing

We consider an approach where the clustering model is mapped into a centralized routing process where path finding and routing table computations are performed by the gateway, where sufficient computation resources are availed for centralized processing with the expectation of achieving an optimized routing configuration. The model uses information collected from the nodes by an energy-frugal distributed signaling protocol.

#### 1.2.4. Distributed Signaling

The centralized routing model described above is complemented by a distributed signaling process where a signaling tree rooted at the sink/gateway is used to: (1) transport the nodes’ information to the sink for topology discovery, path finding and routing table computation; and (2) disseminate the network provisioning information from the gateway to the nodes for routing table configuration. The signaling tree is built using an energy-frugal distributed algorithm.

The works most closely related to ours are found in [11,12,13,14]. The work in [14] considers a node’s service differentiation model similar to ours, but uses a flat topology, which is based on node weight differentiation during path selection. This differs from our model, which is based on hierarchical routing using service-aware clustering. While considering a centralized clustering scheme similar to ours, the works in [11,12,13] are not based on a service awareness/differentiation model similar to ours.

The remainder of this paper is organized as follows: Section 2 describes the energy-efficient IoT model, formulates the service-aware clustering (SAC) problem, presents its algorithmic solution and derives formulas for the optimal number of clusters’ computation. The experimental results are presented in Section 3, while Section 4 contains our conclusions and draws some directions for future work.

## 2. The Energy-Efficient Internet-of-Things Model

**Figure 1:**
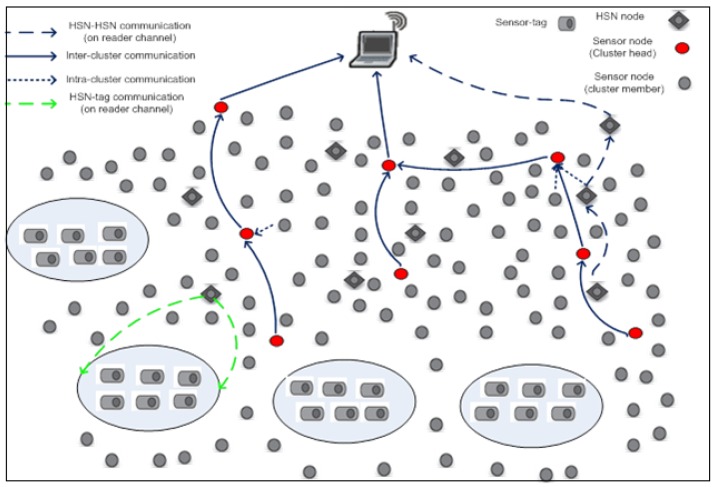
The hybrid Internet-of-Things model. HSN, hybrid sensor node.

As illustrated by Figure 1, a hybrid sensor network is an IoT infrastructure that includes four different types of nodes: (1) conventional sensor node (CSN); (2) sensor tag node (STN); (3) hybrid sensor node (HSN) that combines RFID reader and sensor functionalities; and (4) a base station connected to a gateway (BSN). The CSN nodes and HSN nodes are organized into clusters with each cluster having a cluster head (CH) node that collects, aggregates and forwards the sensor readings from cluster member (CM) nodes, which might be either CSN or HSN nodes to the gateway. On the other hand, the STN nodes located in groups within reading range of an HSN form an IoT island of RFID slave (RS) nodes, which are interrogated periodically by the RFID reader side of the HSN nodes. The HSN nodes that are closer to the base station relay data for other HSN nodes further from the base station. The CSN nodes may be distributed in the network randomly or based on geo-spatial requirements and constraints.

### 2.1. The SAC Architecture: System and Software

The SAC model is based on a system architecture where the STN nodes are assumed to be ultra high frequency (UHF) RFID passive tags, which are outfitted with sensors. These nodes allow tags to be interrogated at a range of up to 10 meters [15], but cannot communicate with each other, even while being energized by the reader due to their passive characteristics. An HSN node integrates both sensor capabilities for sensing what is happening in the environment and identification capabilities to identify numbers from tagged objects or persons. They are endowed with communication interfaces to enable communication with each other and dissemination of the sensor readings to the gateway. The HSN nodes follow a communication model borrowed from [6,16], where two separate channels are used by the hybrid nodes to avoid interference between transmission on the sensor and reader channels. Thus, a 2.4-GHz channel is used by the sensor side of the HSN, while the RFID side uses the 915-MHz channel. The 915-MHz reader channel is assigned to reader-tag communication, while the 2.4-GHz sensor channel is used for communication between HSN and HSN nodes or between an HSN node and its cluster head. By assuming that the micro-controller can differentiate between the different types of data and take appropriate actions that involve decisions on where to send the received data, this results in a multi-layer network where the 2.4-GHz backbone network is layered above 915 MHz, used to carry the STN-to-HSN data. Conventional sensor nodes are also important players of the SAC model, as they can act as relays while providing additional information about their environment. 

The service-aware clustering (SAC) model proposed in this paper is based on a software architecture that maps the sensor nodes’ service delivery into energy awareness and network roles defined by Table 1. The different services delivered by the sensor nodes include sensing by the CSN, sensing + tagging by the STN and sensing + reading by the HSN and aggregation, analysis and processing by the BSN. The different routing roles played by these nodes include cluster head (CH), cluster slave (CS), reader slave (RS), reader master (RM) and base station (BS).

Depending on their service delivery, the different types of nodes and roles may be assigned different energy consumption profiles and levels used to determine the role to be assigned to these nodes during the traffic engineering process. They may be tasked to perform the high-energy consuming roles of the network, for instance, being a cluster head, or the least-energy consuming, such as cluster slave, depending on these profiles and associated energy levels in order to balance the energy dissipation in the network.

**Table 1:** Mapping nodes’ service delivery into routing roles. CSN, conventional sensor node; STN, sensor tag node; BSN, base station node; RS, reader slave; CS, cluster slave; RM, reader master; CH, cluster head.

Node Type	Service Delivery	Energy Consumption Profile	Potential Routing Role
CSN	Sensing	1	CH, CS
STN	Sensing + tagging	2	RS
HSN	Sensing + reading	3	CS, RM
BSN	aggregation + analysis + forwarding	4	BS
**Routing Role**	**Network Service**	**Energy Consumption Level**	**Potential Node**
RS	Identification	1	STN
CS	Sensing	2	CSN, HSN
RM	Reading	3	HSN
CH	aggregation + forwarding	4	CSN, HSN
BS	aggregation, analysis + processing	5	BSN

### 2.2. Radio Model and Energy Budget Computation

**Figure 2:**
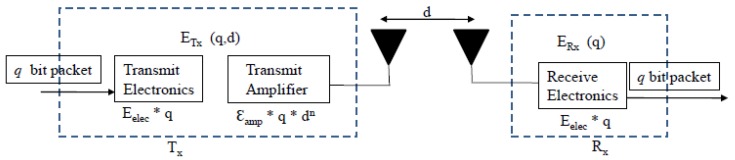
The radio energy model.

Every node in the hybrid IoT network contains a radio communication subsystem that consists of transmitter/receiver electronics, antennae and an amplifier. To determine the energy dissipated by these components, this paper follows the radio energy model discussed in [17] described in Figure 2. In this model, the energy associated with the transfer of *k* bits of data between a transmitter and a receiver node separated by a distance *d* meters is given by Equation (1).

(1) $$\begin{array}{c} \begin{array}{cc} E\; & =E_{Tx}+E_{Rx}\\ E_{Tx} & =E_{elec}*k+E_{amp}*k\\ E_{Rx} & =E_{elec}*k \end{array} \end{array}$$

$\text{where }E_{Tx}$ is the energy spent by a node for transmitting and $E_{Rx}$ is the energy spent by a node for receiving. $E_{elec}$ is the energy needed to run the electronics of the transmitter/receiver, and $E_{amp}$ is the energy required to amplify the transmitted signal. The value of $E_{amp}$ depends on the transmission distance.

Given a threshold transmission distance $d_{0}$, the free space propagation model using parameter $\xi_{FS}$ [11] is employed when $d<d_{0}$ and the two-ray ground reflection model using parameter $\xi_{TR}$ when $d\geq d_{0}$. Using these two models, the energy required to amplify a signal is given by [10].

(2) $$\begin{array}{ccc} E_{amp} & = & \left\{\begin{array}{cc} \xi_{FS}*d^{2} & if\;d<d_{0}\\ \xi_{TR}*d^{4} & if\;d\geq d_{0} \end{array}\right. \end{array}$$

where $d_{0}=\sqrt{\frac{\xi_{FS}}{\xi_{TR}}}$ and the parameters $\xi_{FS}$ and $\xi_{TR}$ were set to $\xi_{FS}=10$ pJ/bit/m${}^{2}$ and $\xi_{TR}=0.0013$ pJ/bit/m${}^{4}$ following [10].

The role that each node performs in the network has a significant impact on the total energy dissipation. Therefore, in order to assign roles fairly, a good understanding of the energy consumption of the different types of nodes is required. This is provided by a radio energy model that describes the energy consumption of each of the hardware components involved in radio communication. Furthermore, a cluster energy budget calculation that considers the energy associated with the different roles played by the system is required. The total energy dissipated by each node is the sum of the energy dissipated for its functions and its role in the network.

- A CSN sensor node acting as CM spends energy on sensing and transmitting data to its cluster head.
- A CSN sensor node acting as CH dissipates energy for receiving, processing and transmitting data from its cluster members, as well as relaying data from other cluster heads.
- An HSN sensor node acting as CM spends energy for sensing and transmitting data to its cluster head, in addition to interrogating sensor tags and relaying data from other HSNs.
- An HSN acting as CH also spends energy for receiving, processing and transmitting data from its cluster members, as well as on interrogating sensor tags and relaying data from other HSNs.

#### 2.2.1. Energy Budget for Sensing Sensor Tags by HSNs

HSNs dissipate a significant amount of energy when interrogating the sensor tags. The total energy dissipated is directly dependent on the density of the sensor tags within the sensing region, which is assumed to be an ellipse, as illustrated in Figure 1. The higher number of sensor tags interrogated, the higher the amount of energy dissipation. Let $S=\{s_{1},s_{2},\ldots ,s_{t}\}$ denote the set of sensor tags in the network and $H=\{h_{1},h_{2},\ldots ,h_{t}\}$ the set of HSNs within reading range of the sensor tags. Then, let $S_{i}$ denote a subset of *S* that represents the set of sensor tags randomly distributed within the reading area of $h_{i}$. In a given region, the set $S_{i}$ can only have a maximum of *t* sensor tags. Each sensor tag generates *k* bits of data. Assuming that $h_{i}$ successfully receives the data from all *t* sensor tags, then by following the principle in Equation (1), the energy consumption of $h_{i}$ for interrogating *t* sensor tags would be:

(3) $$E_{i}=\sum_{j=1}^{t}[\left(E_{elec}*k+\xi_{amp}*k*d_{i,j}^{\alpha }\right)+E_{elec}*k]$$

where $\xi_{amp}\in \left\{\xi_{FS},\xi_{TR}\right\}$ and $0<d_{i,j}\leq max\left\{d_{1},\ldots ,d_{t}\right\}$ is the distance between $h_{i}$ and sensor tag *j* within its reading range, while *α* is the propagation loss coefficient with values of two or four, depending on the value of *d*, as defined in Equation (2).

#### 2.2.2. Energy Budget for Data Aggregation by Cluster Heads

One of the advantages of organizing the WSN into clusters is that the amount of data transmitted to the base station can be compressed and correlated. To determine the energy consumption of a cluster head for aggregating data, we use the following principle.

Let $E_{DA}$ denote the total energy dissipated by a cluster head node’s digital electronics for aggregating *k* bits of data from *m* cluster members. Then,

(4) $$E_{DA}=\left(C_{tot}*V_{DD}+I_{leak}*\Delta t\right)V_{DD}$$

This energy is the sum of the energy lost to switch capacitance ($C_{tot}$) and the energy lost in current leakage $I_{leak}$ [13]. $V_{DD}$ is the voltage supply, and Δt is the latency for aggregating *k* bits of data from each *m* cluster members. Using the experimental results of [18] and the parameters in [10], then the value of $E_{DA}$ used in this paper is 5 nJ/bit/signal.

#### 2.2.3. Total Energy Dissipation in a Cluster

The sensor nodes of the WSN can be further divided into cluster heads and cluster members. Using Equations (2)–(4), we calculated the total energy dissipated by a sensor node based on its role in the network. The total energy dissipated by a sensor node acting either as cluster member or a cluster head (CH) of *m* cluster members which also relays the data of other *l* cluster heads is given by Equation (5). Note that for a node acting as a cluster member (CM), the total energy dissipated is the sum of sensing and communication energies. Therefore, a sensor node spends in total:

(5) $$\begin{array}{cc} E_{tot} & =\left\{\begin{array}{cc} \left(m+l\right)E_{rx}^{CH}+E_{DA}+E_{tx}^{CH}; & \;if\;CH\\ E_{tx}^{CM}+E_{sens}^{CM}; & \;if\;CM \end{array}\right. \end{array}$$

where $E_{tx}^{CH}$ is the energy used by a cluster head while transmitting data to the next cluster head or base station and $E_{rx}^{CH}$ is the energy dissipated for receiving data from a cluster member. $E_{tx}^{CM}$ is the energy used while transmitting data inside the cluster to a cluster member, and $E_{sens}^{CH}$ is the energy spent for sensing the environment.

### 2.3. The Service-Aware Clustering Model

The efficiency of an IoT traffic engineering model depends on its routing configuration, which usually reveals the actual routing paths followed by the sensor readings from their environment to the base station during operation. An efficient routing configuration is obtained by engineering the traffic flows carrying the sensor readings to route these readings from their controlling locations to the base station with the expectation of optimizing an objective function (maximizing a reward function or minimizing a penalty function) subject to some constraints on routing metrics, such as the delay, reliability or energy consumption.

#### 2.3.1. The Service-Aware Clustering Problem

Let us consider a wireless sensor network represented by a directed graph G=(N,L,s), where N is the set of sensor nodes, L is the set of wireless links between nodes and *s* is unique and selected as the network base station. Assume that $R\left(G\right)=\left\{R_{i}\right\}$ is the set of routing configurations associated with the network G where each of the routing configurations $R_{i}$ is expressed by the tuple $R_{i}=\left(N_{c},N_{n},L_{c},L_{n}\right)$ where $N_{c}$ and $N_{n}$ are the set of cluster heads, including the base station *s* and normal nodes, respectively, while $L_{c}$ is the set of links connecting the cluster heads that form a backbone for the sensor readings to the base station and $L_{n}$ is the set of links connecting the cluster heads to cluster member nodes. Assume a service-aware model, where each node *x* is allocated a service delivery level S(x). The SAC problem consists of finding an efficient routing configuration $R_{ef}\in R\left(G\right)$, such that:

(6) $${\hat{\tau }}_{egy}\left(R_{ef}\right)=\max_{R_{i}\in R\left(G\right)}{\hat{\tau }}_{egy}\left(R_{i}\right)\;$$

subjectto

(6a) $$\begin{array}{cc} P_{r}\left(x\in N_{c}\right) & \geq P_{r}\left(y\in N_{c}\right)\;\mathrm{if}\;\tau_{egy}\left(y\right)<avgE\leq \tau_{egy}\left(x\right) \end{array}$$

(6b) $$\begin{array}{cc} P_{r}\left(x\in N_{c}\right) & \geq P_{r}\left(y\in N_{c}\right)\;\mathrm{if}\;S\left(x\right)\leq S\left(y\right)\;\forall x,y\in N \end{array}$$

(6c) $$\begin{array}{cc} P_{r}\left(x\in N_{n}\right) & \geq P_{r}\left(y\in N_{n}\right)\;\mathrm{if}\;S\left(x\right)\geq S\left(y\right)\;\forall x,y\in N \end{array}$$

(6d) $$\begin{array}{cc} j\in N[x] & \;\mathrm{if}\;D_{is}\left(j,x\right)<D_{is}\left(i,x\right)\;\forall i,j,x\in N_{c} \end{array}$$

(6e) $$\begin{array}{cc} j\in N[x] & \;\mathrm{if}\;D_{is}\left(j,x\right)<C_{ov}\;\forall i,j,x\in N \end{array}$$

where N[x] is the set of neighbors of node *x* and $D_{is}\left(j,x\right)$ is the Euclidean distance between nodes *j* and *x*; ${\hat{\tau }}_{egy}\left(R_{ef}\right)$ represents the residual energy of a routing configuration, while $\tau_{egy}\left(x\right)$ expresses the residual energy of a single node *x*; and $N=N_{c}\cup N_{n}$. The average residual energy avgE of all of the nodes of a network N is expressed by $avgE=\frac{\sum_{y\in N}\tau_{egy}\left(y\right)}{|N|}$.

Note that, as formulated above, the SAC problem expresses:

- Energy-frugal routing expressed by the routing objective Equation (6), where the most energy-efficient routing configuration is the one with the maximum residual energy.
- Energy-aware clustering expressed by the constraint Equation (6a) used in the energy-aware pre-selection phase of the cluster head pre-election process described in Section 2.4. This constraint is used to assign cluster head roles to the nodes depending on the average sensor network residual energy.
- Service-aware clustering expressed by the constraint Equation (6b,c) used in the service-aware pre-selection phase of the cluster head pre-election process described in Section 2.4. These constraints are used to assign cluster head or cluster member/slave roles to the nodes depending on their service level.
- Neighbor selection on the multi-hop backbone network of cluster heads as expressed by the distance minimization constraint in the Equation (6d) and the neighbor selection and association constraint based on the wireless communication range in Equation (6e).

By using a probabilistic approach where service and energy awareness are expressed by probabilities of nodes becoming cluster heads or members depending on their service levels and residual energy, the SAC formulation enables striking a good balance between service awareness and energy awareness: e.g., while HSN nodes should naturally be excluded from becoming CH because they are service intensive, there might be cases during a WSN operation where they could still qualify to become the CH when they are more battery energized than CSN nodes.

#### 2.3.2. The Service-Aware Clustering Solution

**Figure 3:**
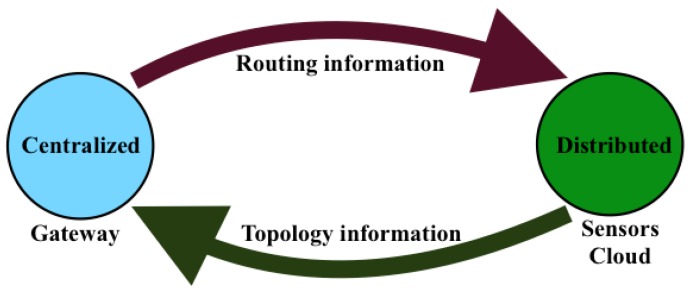
The IoT network management model.

As formulated above, many of the constraints of the service-aware clustering problem and its objective function involve global parameters that may be processed by only a centralized processing entity that has a global view of the sensor network. Therefore, the SAC formulation falls in the category of centralized problems, which require a centralized algorithmic solution, like LEACH-C, MOCRN and DECSA. However, SAC differs from these algorithms by the service awareness constraints expressed by Equation (6c,d). In a WSN, the gateway is a natural fit for such centralized processing, as it is usually attached to a stable power supply and has enough processing and storage capabilities for a centralized process. However, the centralized process proposed in this paper needs to be complemented by a distributed network provisioning process enabling local routing tables on sensor nodes to be configured to reflect the efficient routing configuration $R_{ef}$ computed by the centralized process in the gateway. The resulting IoT network management model is depicted by Figure 3.

**Figure 4:**
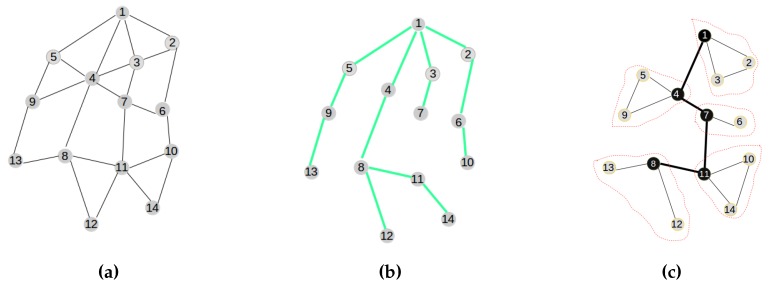
The hybrid traffic engineering model. (**a**) Initial network; (**b**) The signaling tree; (**c**) The clustered topology.

The integration of centralized clustering and distributed provisioning resulting from the IoT network management model of Figure 3 is illustrated by the hybrid traffic engineering model depicted by Figure 4 where the signaling tree in Figure 4b built by the distributed process from the network topology in Figure 4a is used to ferry signaling messages to the gateway for building the clustered network topology of Figure 4c by the centralized process.

The efficiency of the SAC solution proposed in this paper is based on a good distribution of routing roles to sensor nodes in order to minimize the overall energy dissipation in the sensor network. The SAC solution adopted in this paper is built around the following key features:

1. Centralized clustering: The clustering process is led by the gateway attached to the base station rather than the sensor nodes for reasons described above and in order to avoid the energy wastage associated with a distributed model, enabling the sensor nodes to communicate amongst themselves to elect their cluster heads and build their clusters. Figure 4c depicts the clustered network configuration resulting from the initial network in Figure 4a.
2. Distributed provisioning: As suggested above, a distributed process is needed for the sensor network provisioning. It is implemented with the objective of building a signaling tree for the sensor nodes’ information discovery by the gateway and sensor nodes’ role confirmation by the gateway. Figure 4b depicts the signaling tree resulting from the initial network configuration of Figure 4a.
3. Optimal number of clusters: The algorithm works with a number of predetermined clusters to control the size of messages routed between and within clusters. Such a number might be related to the size of the sensor network, as well as some geo-spatial constraints and/or based on an optimal number of clusters’ computation dictated by energy efficiency requirements.
4. Energy-aware routing: The algorithm assumes that the initial and residual node energy might be known at any moment by using the signaling tree to propagate topology information to the gateway for path computation and network provisioning information to the nodes for routing table construction.
5. Service-aware clustering: As assumed earlier, the energy profiles of nodes are related to their types and their service delivery types: service-intensive nodes will consume much energy during operation, while energy-frugal nodes will consume less energy during operation. This is taken into consideration by the algorithm through service differentiation.

##### Distributed Provisioning

**Figure 5:**
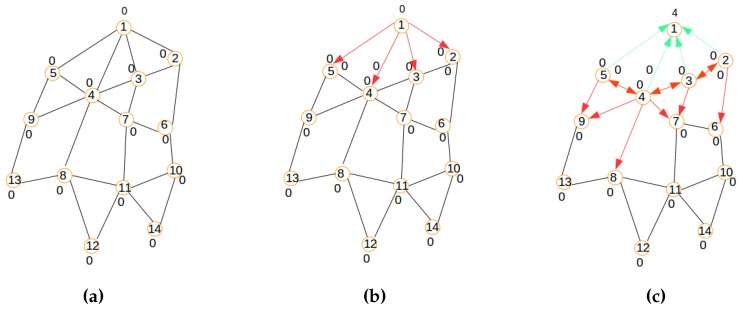
Signaling process: Steps 1, 2 and 3. (**a**) Initial network; (**b**) Step 2; (**c**) Step 3.

**Figure 6:**
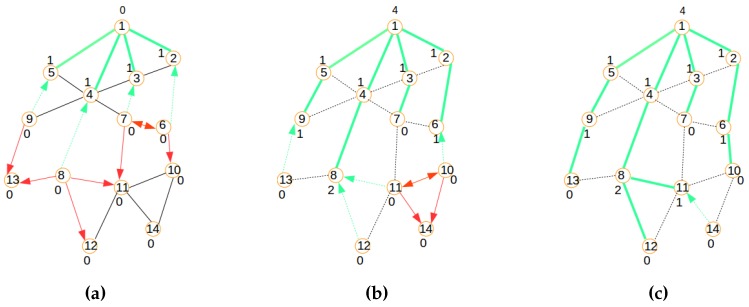
Signaling process: Steps 4, 5 and 6. (**a**) Step 4; (**b**) Step 5; (**c**) Step 6.

**Figure 7:**
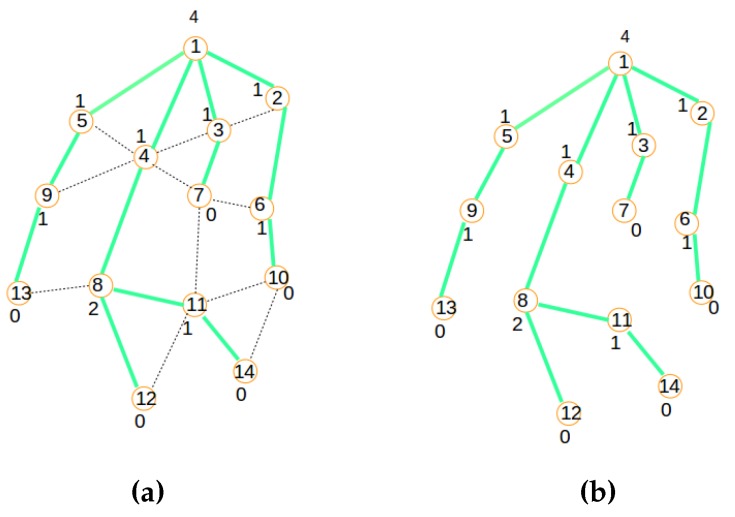
Signaling process: Steps 7 and 8; (**a**) Final tree; (**b**) Signaling tree.

A modified version of the LIBPprotocol [19,20] is used in this paper to build the signaling tree required for network provisioning. Its main steps consist of building a routing tree for the signaling messages, as illustrated by Figure 5, Figure 6 and Figure 7. The main steps of the signaling tree construction algorithm are described as follows:

- Initialization: The routing process starts with an initial network depicted by the graph in Figure 5a where Node 1 is the sink.
- Beaconing process: As shown in Figure 5b,c and Figure 6a,b a beaconing process is initiated by a sending node, which announces to the receiving node the intention of the sender of becoming the parent of the receiver. In all figures, the beaconing process is identified by red arrows. This beacon message will reach only a set of neighbors j∈N[s] of a sender (*s*) that meet the constraint $D_{is}\left(s,j\right)<C_{ov}$ expressing that the distance between the sender *s* and any of its neighbors *j* should be inferior to the wireless coverage range $C_{ov}$. While Step 1 shows the initial connected graph of the network, Step 2 reveals the initial beaconing process. Step 3 reveals the initial acknowledgment and forwarding of the beaconing process.
- Acknowledgment and relaying: In the steps in Figure 5c and Figure 6a,b,c, acknowledgments are illustrated by green arrows, which reflect the selection of a parent node chosen among the nodes that sent a beacon message. In contrast to the LIBP protocol where the acknowledgments are used to select a parent among many candidates, the acknowledgment in the SAC protocol is used to both select a parent and to carry a node’s information to the sink/gateway. Note that while all neighbors of the sink have a unique parent selection choice (the sink), the other different nodes select their unique parent from different potential originators of the beacon message. Steps 3, 4 and 5 show how the acknowledgment (in green arrows) and beaconing (in red arrows) processes are recursively repeated, with the expectation of having the beacons reaching the rest of the network, as well as acknowledgments sent back to the root for building a routing topology for the clustered network configuration computation.
- Weight: The least interference paradigm maps the acknowledgment process into a weight setting by: (1) having each node receiving only one acknowledgment from each of its potential children; and (2) the sum of received acknowledgment messages representing a weight on a node expressing the interference on the node of the paths carrying the sensor readings from sensing locations to the sink.
- Termination criteria: The beaconing process is repeated recursively using a breadth-first approach, resulting in the formation of a spanning tree when the beacons reach the leaf nodes, which are marked with weight zero, as they do not receive any acknowledgment message from any node, as expressed by Figure 7a. In such a tree, each node will exhibit a weight corresponding to the number of children it has.

##### Centralized Clustering

The service-aware clustering proposed in this paper consists of mapping a physical network consisting of normal and hybrid nodes into a logical topology containing cluster heads and cluster slave/member nodes. It follows the five steps described below

- Step 1: This step consists of applying the distributed provisioning described above to allow the gateway to get the necessary information from the sensor nodes in order to achieve global computation. The node discovery process is piggy backed on the signaling tree construction, which will produce a routing tree used later in the network provisioning phase.
- Step 2: In this step, clusters are constructed based on the information received from the sensor nodes. This step is executed following three main sub-steps: (i) average residual network energy calculation used to achieve energy-aware pre-selection of cluster heads, while the sensor nodes’ service types reported to the gateway are used to achieve service-aware pre-selection; (ii) cluster head election achieved by comparing the number of pre-selected cluster heads to the required number of cluster heads; (iii) cluster head-slave association performed using an optimization method that considers both the geographical location of nodes and their residual energy; and (iv) TDMA schedule generation to preclude an interference-free transmission of the information (sensor readings).
- Step 3: A node’s role confirmation is achieved by using the signaling tree built by making use of the distributed provisioning process described earlier. It aims at informing the nodes of their roles, the next hop to the gateway and also their TDM slots.
- Step 4: During this step, the sensor backbone of cluster heads is constructed by using the highest signal strength - an approach that translates into increasing the probability of selecting the nearest neighbor’s node and, thus, leading to the shortest delays on the backbone.
- Step 5: This step uses the TDMA schedule resulting from Step 2 to enable sensor readings’ dissemination from the nodes to the gateway.

### 2.4. The SAC Algorithm

Our proposed routing algorithm operates in five steps to elect the best cluster head. Each step is then repeated after a round of *T* seconds. The five steps are described below.

#### 2.4.1. Step 1: Nodes’ Discovery

During this step, each sensor node (HSN and CSN) receives a beacon message carrying a query from the base station and signals its presence in the network by selecting a potential parent (next hop to the base station) and replying to the query by sending to its parent node a hello packet containing its identification number (ID) and service type (SE), coordinates ([X,Y,Z]), initial energy (IE) at start-up of the routing process and, later, its residual energy ($E=\tau_{egy}\left(ID\right)$) during operation. Note that in our problem formulation, SE=S(ID) and $E=\tau_{egy}\left(ID\right)$.

#### 2.4.2. Step 2: Cluster Formation

**Figure 8:**
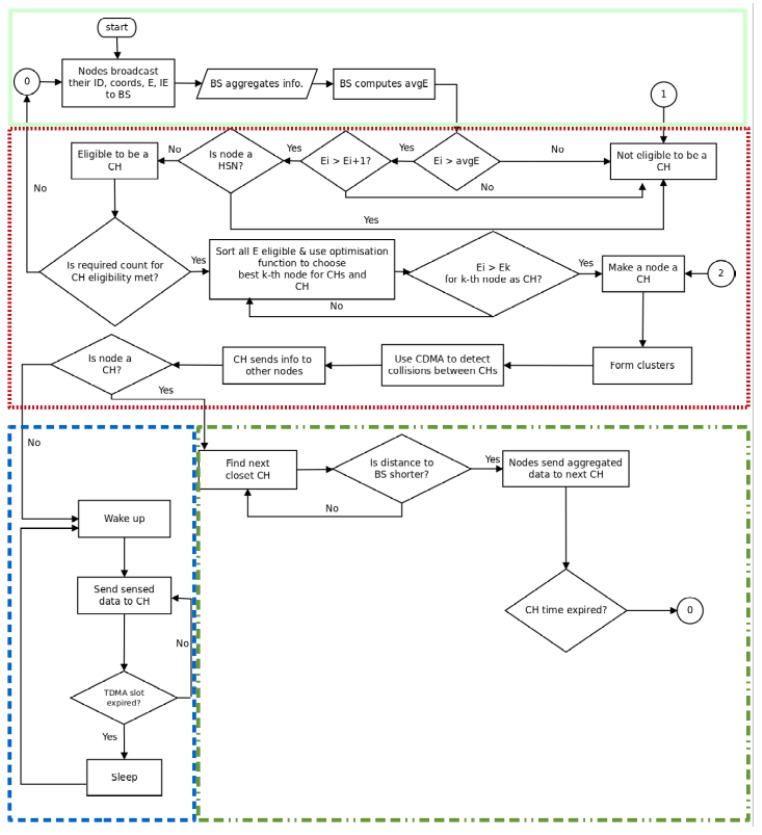
The service-aware clustering (SAC) algorithm.

During this step, the base station arranges the sensor nodes into an optimized number of clusters. The steps taken by the base station in organizing the network into clusters are presented below. Figure 8 summarizes the process that each node undergoes to contribute to the cluster head election.

1. Cluster head pre-election: After receiving the broadcast information from all nodes, the base station uses the residual energy information to compute the network’s average residual energy $avgE==\frac{\sum_{y\in N}\tau_{egy}\left(y\right)}{|N|}$ for all nodes $x\in N=N_{c}\cup N_{n}$. Knowing the network’s residual energy, the base station proceeds with the pre-election of cluster heads. Two processes are used in cluster head pre-election.
  Energy-aware pre-selection: First, the base station compares the energy of the current node to the average network’s residual energy. If the node energy is higher, then the current node energy is compared to that of the next node in the list. If it is still higher, then the current node is a candidate for the cluster head election. In agreement with Equation (6a) and the nodes’s roles defined in Table 1, the combination of both of these conditions enables the SAC algorithm to increase the possibility of electing as cluster head only those nodes that have the highest energy in the network.
  Service-aware pre-selection: In the second stage of the pre-election, the base station uses the nodes’ service (SE) to check if the selected node is an HSN. This is because, following the constraint Equation (6c) of the SAC formulation, we want to prevent HSNs from being elected as cluster heads. Here, we assume that using the nodes’ SE, the base station is able to distinguish HSNs from other nodes.
2. Cluster head election: Finally, the election process is concluded by checking if the number of nodes that have met the previous requirements are less than the pre-determined number of cluster heads. If this is the case, then the base station re-examines each node in the network and selects any node whose energy is greater than the average.
3. Cluster slave association: After the base station has determined all the eligible nodes, it performs an optimization algorithm that uses the geographical location of each candidate and its current residual energy to elect only the best *k* cluster heads and clusters that optimize the Equation (7).
  (7) $$\begin{array}{ccc} cost & = & \beta f_{1}\times \left(1-\beta \right)f_{2}\\ f_{1} & = & max\{\sum_{\forall n_{i}\in C_{k}}\frac{d(n_{i},CH_{k})}{|C_{k}|}\}\\ f_{2} & = & \frac{\sum_{i=1}^{N}E\left(n_{i}\right)}{\sum_{k=1}^{K}E\left(CH_{k}\right)} \end{array}$$
  Where $f_{1}$ represents the maximum of the mean distance between non-eligible nodes and their associated cluster heads, $f_{2}$ the ratio of the total initial energy of all nodes over the total residual energy of the temporary elected cluster heads in the current round and $c_{k}$ the number of nodes that belong to cluster $C_{k}$ for k∈{1,2,…,K}. Finally, constant *β* is used to weigh the contribution of each of the functions.
  The two functions $f_{1}$ and $f_{2}$ provide the base station with the ability to evaluate the fitness level of each individual node $n_{i}$. Node $n_{i}$ is associated with a cluster head $CH_{k}$ only if its distance to $CH_{k}$ is the smallest of its distances to all other cluster heads. With this, the intra- cluster distance between cluster heads and their members and the network’s energy usage are both optimized.
4. TDMA transmission schedule: After cluster formation, the base station allocates time slots to each node within a cluster using time division multiple access (TDMA) while code division multiple access (CDMA) is used to reduce collision between cluster heads.

#### 2.4.3. Step 3: Node Roles’ Confirmation

Finally, the base station informs the nodes about their roles in the network by using the signaling tree built during node discovery to broadcast roles and association messages. Using this process, each node is assigned its role in the network (CH or CM) and its next hop to the base station. The message content will be of the form [ID,RL,NH,ST], where ID, RL, NH and ST represent, respectively, a node’s ID, its role in the network (CM or CH), its next hop to the base station and its time slot for transmission. For each message received, a node is made aware of its role and next hop to the base station if the message ID of a node matches the ID field of the signaling message sent by the base station and, thus, adjusts its forwarding table to reflect the information received from the base station.

#### 2.4.4. Step 4: Backbone Construction

This stage determines how the multi-hop routes are set up by the cluster heads to create a sensor network backbone. The cluster heads use their radio to create the sensor backbone as follows.

- In order to optimize the routing process, each cluster head uses the closest cluster head as the next hop to the base station, as determined by the signal strength emitted by all neighboring cluster heads, including the base station.
- Each cluster head selects the strongest signal strength neighbor as its next hop to the gateway and adjusts its routing table accordingly by recording the IDs of its pre-hop node and next-hop node. Note that as currently designed, this backbone could lead to a tree-like or mesh topology.

#### 2.4.5. Step 5: TDMA Communication

In this paper, we assumed that each sensor node senses data at a fixed rate and always has data to send to its cluster head during its allocated time slot. Then, the node switches into sleep mode until the next transmission slot. Once data is received from cluster members, it is aggregated by the cluster head and then forwarded to the next cluster head along the path to the base station. The HSNs use the 915-MHz channel periodically to interrogate sensor tags within their range, while the 2.4-GHz channel is used for intra-cluster communication. The RFID reader operates differently, that is once the information is collected, it is routed to the next hop along the HSN-to-HSN path.

## 3. Performance Evaluation

Different experiments were conducted to compare the performance achieved by the SAC protocol/algorithm to the family of LEACH protocols/algorithms (LEACH and LEACH-C) and more recent clustering protocols/algorithms, DECSA and MOCRN. The experiments were conducted in a realistic sensor network environment where distributed signaling using Contiki/Cooja and centralized clustering were run in two different threads, but communicating through a sink interface: a SAC thread tasked to execute the SAC algorithm and report to the Cooja thread for routing table set up and a Cooja thread tasked to achieve distributed signaling and packet forwarding. It then reports to the SAC thread for routing configuration computation. Therefore, the experimental sensor network environment used in this paper mimics the IoT network management model in Figure 3, but with the routing and topology information being exchanged between the cloud of sensor nodes and the gateway through the sink interface. The centralized simulative environment was designed based on the different formulas described in the SAC model and the parameters of the hybrid IoT architecture in Section 2, while the distributed signaling was emulated in a Contiki/Cooja environment using the experimental setup described by Table 2.

**Table 2:** Experimental setup: Cooja parameters.

Test Attributes	Test Value
Beacon Interval	30 s (LIBP), Adaptive (CTP, RPL)
Messaging Interval	30 s, but following the TDMA schedule computed by SAC
Message Contents	“Hello from node”
Simulation Runtime	1000 s, but with 120 s for network self-organization
TX/INTRange	50 m/100 m

Table 2 above outlines the experiment runtime. In short, unless otherwise specified, the networks are each given a 120-s period to allow for the network to settle; thereafter, the network is run for eight minutes to give a total simulation runtime of 1000 s. Each node will periodically send a packet containing the string “Hello from node” as its packet data. Since each node is given 880 s to send the data at a period of 30 s, the nodes will each send 29 packets of data to be collected by the sink. For the various experiments, all of Cooja’s existing profiling tools were used as experimentation tools. Emulation timers and node real-time timers were used as experimentation tools for time-sensitive experiments. For discerning between control plane traffic and data plane traffic, the packets were flagged accordingly. The packets would then trigger a counter, which would hold a value that shows how many times a packet of that particular classification occurred as traffic during simulation runtime. For some of the experiments, one single network was considered, and for others, several networks of different sizes were considered with the objective of evaluating the scalability of the different protocols/algorithms. For all networks, we considered 80% of normal nodes (CSN nodes) and 20% of hybrid nodes (HSN nodes). For each beacon interval, called the “epoch”, a high level description of the sensor network environment mimicking the interplay between the centralized and distributed provisioning processes illustrated by Figure 3 is briefly described as follows: 

For each epoch:

1. Using the Contiki/Cooja environment, build the signaling tree.
2. Upload topology to the gateway through the sink interface.
3. Compute the efficient routing configuration $R_{ef}$ using centralized clustering.
4. Download the routing information in the sensor nodes through the signaling interface.
5. Build forwarding tables on the nodes based on the provisioning information received through distributed signaling.
6. Emulate data forwarding in the Contiki/Cooja environment and record statistics.

### 3.1. The SAC Protocol Performance

Objective: This experiment was conducted to assess the relevance of using the SAC model in IoT settings by comparing it to a breadth-first search (BFS) and a service-blind clustering (SBC) algorithm. The BFS algorithm is a non-hierarchical routing algorithm that finds routing paths for the sensor readings by considering a flat network topology, while the SBC is a hierarchical routing algorithm that is based on only signal strength for cluster selection and cluster head-member association, discounting energy and service awareness. This experiment was complemented by another experiment aiming at comparing two different versions of the SAC algorithm: (1) the SAC algorithm where over the experimental time, energy awareness and service awareness are combined to find efficient routing paths; and (2) a dual-mode SAC algorithm called SAC-MIX, where energy awareness is implemented during the first half of the experiment when all nodes are assumed to have enough residual energy. Service awareness is implemented during the second half of the experiment when the nodes are assumed to have depleted enough energy.

Network: Two networks were considered: (1) a hybrid network consisting of 100 nodes randomly distributed within the sensing area; and (2) a conventional sensor network consisting of 100 CSN nodes. The hybrid sensor network consisted of 80 CSN nodes and 20 HSN nodes, where each HSN node consumes 0.5 J more than the energy of a CSN node. The hybrid sensor network running SAC had a total initial energy of 110 joules, while the normal sensor network running the BFS and SBC had a total initial energy of 100 joules. This is because, in practice, the HSN nodes are initially more energized than the CSN nodes.

**Figure 9:**
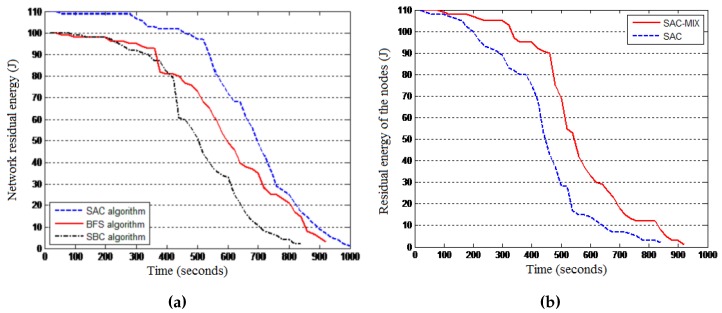
SAC performance. (**a**) SAC, breadth-first search (BFS) and service-blind clustering (SBC); (**b**) SAC-MIXand SAC.

Results: The results presented by Figure 9a revealed the relative efficiency of the SAC algorithm compared to both BFS and SBC in terms of residual energy. The results also reveal that through implementing hierarchical routing, a service-blind algorithm can be outperformed by a non-hierarchical algorithm. Figure 9b reveals that SAC-MIX outperforms SAC. Therefore, SAC-MIX will be considered in the rest of our experiments and referred to as SAC.

### 3.2. The Relevance of Service Awareness

Objective: This experiment was conducted to assess the relevance of service awareness by comparing the SAC algorithm with the family of energy-aware LEACH algorithms in terms of energy dissipation per round of the experiment and total energy dissipation for the whole experiment. LEACH and LEACH-C were considered for these experiments.

Network: Two network models were used in this experiment: a 50-node network and a 250-node network each with 80% of CSN nodes and 20% of HSN nodes randomly distributed in a 100 m × 100 m sensing area. The initial energy of each node was 1 J, and the simulation time (round) varied depending on the experiment. The energy dissipation of the nodes was taken every 10 s.

**Figure 10:**
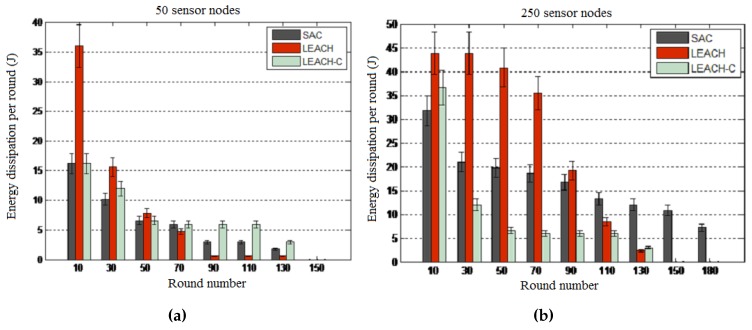
Energy consumption per round. (**a**) 50-node network; (**b**) 250-node network.

**Figure 11:**
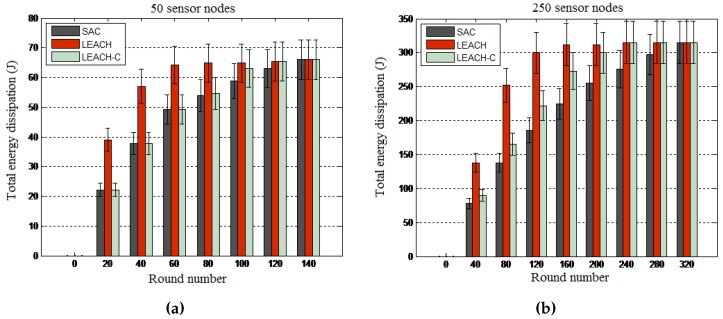
Total energy consumption. (**a**) 50-node network; (**b**) 250-node network.

Results: The results presented in Figure 10 and Figure 11 reveal that SAC outperforms both LEACH algorithms in terms of energy consumption per round and total energy consumption. By showing that LEACH performs worse than both the SAC and LEACH-C algorithms, these results reveal the optimality of centralized routing processes where the central node uses global knowledge to compute more optimized network configurations. Note that though the total energy consumption is the same for all algorithms at the end of the experiment, in general, the SAC algorithm performs better than the two other algorithms when considering the energy consumption per round of the 10-s interval.

### 3.3. Sensor Network Lifetime: First and Last Node to Die

Objective: This experiment was conducted to evaluate the network lifetime when defined in terms of the first node to die (FND) and the last node to die (LND) and the relative efficiency of the different protocols in terms of the number of nodes alive during the simulation period.

Network: The network model used consisted of 100 nodes randomly distributed in a 100 m × 100 m sensing area with 80 normal nodes and 20 hybrid nodes, where each hybrid node consumes 0.5 J more than the energy of a normal node. The initial energy of each node is 1 J, and the simulation time (round) runs for 1000 s. The average energy consumption of the nodes is taken every 100 s. The receiving and sending power of each node is 0.360 W and 0.395 W. The same parameters are used for all of the protocols. Two network configurations with the base station located differently were considered in order to evaluate the impact of the communication distance between the nodes and the base station on the network lifetime: one configuration with the base station located at the center of the network area and another one with the base station located outside the network area.

**Figure 12:**
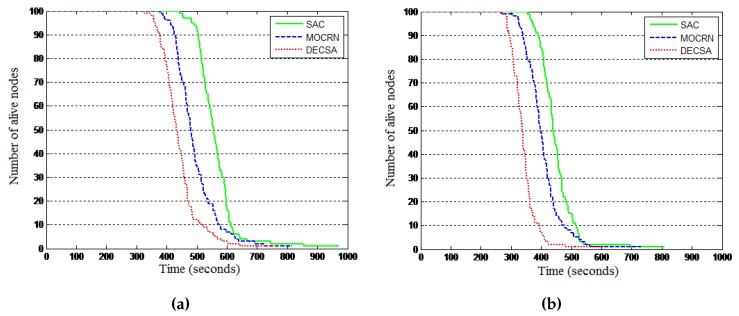
Energy consumption. (**a**) Alive nodes: BS at the center; (**b**) Alive nodes: BS is outside.

Results: The results are presented in Figure 12 and Table 3. Figure 12 reveals that in general, the three protocols dissipate their energy using a similar pattern for both network configurations, with DECSA being the most energy-hungry protocol, followed by MOCRN and, lastly, SAC. The results in Table 3 reveal a similar energy dissipating pattern for the LND and FND performance parameters. It can be seen from the table that when the base station is first placed at the center of the network, the first nodes die (FND) at the 332th round in DECSA, 391th round in MOCRN and at the 448th round in SAC. Similarly, the last nodes die (LND) at the 728th round, 831th round and 968th round in DECSA, MOCRN and SAC, respectively. It can be observed that with the base station located at the center of the network, the difference between the lifetimes of the three protocols is not significant due to the short communication distance between nodes and the base station. Thereafter, the base station was placed outside the network area at coordinate (50,180) m. Table 3 revealed in this case that the first nodes died at the 255th round in DECSA, at the 298th round in MOCRN and at the 361th round in SAC, while the last nodes died at the 556th round, the 746th round and the 802th round in the DECSA, MOCRN and SAC protocols, respectively. The two network configurations revealed the relative efficiency of SAC compared to DECSA and MOCRN. Such relative efficiency is a result of both service awareness and the application of a CH-to-CM cluster association model, where SAC uses the received signal strength (RSS), while MOCRN uses the hop count and DECSA the distance.

**Table 3:** Network lifetime: first node to die (FND) and last node to die (LND).

BS at the Center	DECSA	MOCRN	SAC
FND (round)	332	391	448
LND (round)	728	831	968
**BS Outside**	**DECSA**	**MOCRN**	**SAC**
FND (round)	255	298	361
LND (round)	556	746	802

It can be seen from the table above that SAC runs more rounds before the first and last node dies. The reason is that our algorithm considered parameters such as the current energy of the sensor nodes, service differentiation and the distance between the nodes during the election of new cluster heads. Hence, it performs better than the other two protocols.

### 3.4. Sensor Network Lifetime: Average Lifetime and Scalability

The sensor network lifetime $t_{N}$ can be defined as the sum of the initial energy of all sensor nodes divided by the total energy dissipated per node. It can be expressed as follows:

(8) $$t_{N}=\frac{\sum_{i=1}^{N}E_{i}}{E_{tx}^{CM}+E_{sens}^{CM}+\left(m+l\right)E_{rx}^{CH}+E_{DA}*k*m+E_{tx}^{CH}}$$

where $E_{i}$ is the initial energy of each sensor node and Nis the number of sensor nodes in a network. The lifetime of a normal sensor node $t_{CM}$ and the lifetime of a cluster head node $t_{CH}$ are given as follows:

(9) $$\begin{array}{c} \begin{array}{cc} t_{CM} & =\frac{E_{i}^{CM}}{E_{tx}^{CM}+E_{sens}^{CM}}\\ t_{CH} & =\frac{E_{i}^{CH}}{\left(\left(m+l\right)E_{rx}^{CH}+E_{DA}*k*m\right)+E_{tx}^{CH}} \end{array} \end{array}$$

Objective: We conducted a set of experiments to evaluate the average lifetime of the sensor networks of different sizes ranging from 100 nodes–500 nodes. We also evaluated the time till the last node dies (LND) for the same network configurations and the scalability of the algorithms when applied to a 500-node network and when scalability is expressed in terms of the optimal number of clusters.

Network: Sensor networks of different sizes ranging from 100–500 nodes were considered for these experiments with the base station located at the center of the network.

**Figure 13:**
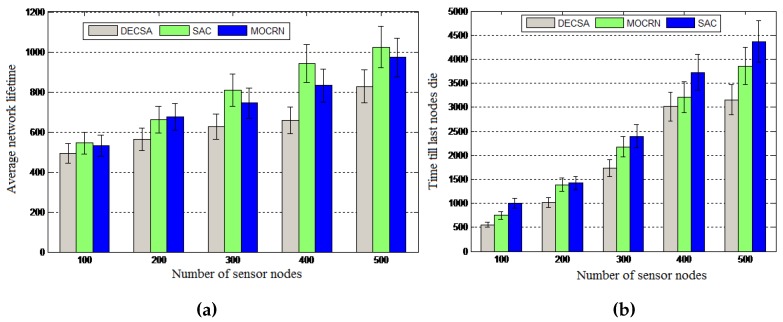
Average network lifetime. (**a**) Average network lifetime; (**b**) Last node to die (LND).

Results: The results presented in Figure 13a reveal that the average network lifetime achieved by SAC for 100 sensor nodes is 10.8% higher than DECSA and 2.4% higher than MOCRN. Moreover, SAC’s average lifetime was 23.7% higher than DECSA and 5.3% higher than MOCRN when the number of nodes was increased to 500. The reason is that sensor nodes are able to communicate with their cluster heads through a short distance due to the uniform distribution of cluster heads within the network. Finally, the method used for the selection of cluster heads considered the node location and the residual energy of the nodes, while DECSA selects cluster heads based on residual energy only, and MOCRN is based on distance. A similar performance pattern was revealed by Figure 13b for the last node to die, showing that SAC outperforms the two other algorithms.

The optimal number of clusters is another important parameter, which can be determined by deriving the total energy consumed in the whole network $E_{total}=KE_{cluster}$ with respect to the the number of clusters *K*. In our case, the energy consumed in a cluster consists of the energy consumed by the cluster head $E_{H}$, and the member nodes $E_{N}$ in a given round is given by $E_{cluster}=E_{H}+\left(C_{k}-1\right)E_{N}$, while the total energy consumed in the whole network is expressed by $E_{total}=KE_{cluster}$. Assuming that $E_{total}$ is the total energy cost for sensor nodes to transmit a bit of data to their respective cluster heads and $E_{F}=\frac{E_{DA}}{k}$ the energy dissipation for aggregating one bit of data packets, the total energy $E_{total}$ will be expressed by:

(10) $$\begin{array}{ccc} E_{total} & = & 2\left(V-K\right)E_{elec}+\left(V-K\right)\epsilon_{fs}\frac{M^{2}}{6K}+VE_{F}+KE_{elec}+K\epsilon_{amp}d_{toBS}^{4} \end{array}$$

The optimal number of clusters is determined by setting the derivative of $E_{total}$ with respect to K to zero. This gives:

(11) $$\begin{array}{c} \begin{array}{cc} K^{2} & =\frac{V\epsilon_{fs}M^{2}}{6\epsilon_{amp}d_{toBS}^{4}}\\ K_{opt} & =\sqrt{\frac{V\epsilon_{fs}M^{2}}{6\epsilon_{amp}d_{toBS}^{4}}} \end{array} \end{array}$$

Therefore, the value of $K_{opt}$ in Equation (11) is the optimal number of clusters for our energy model.

**Figure 14:**
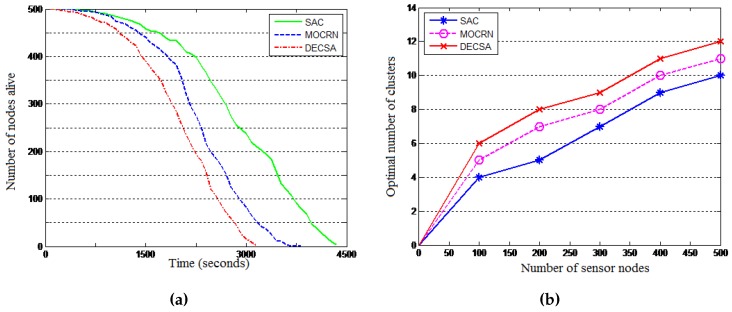
Scalability of the SAC algorithm. (**a**) Number of nodes alive; (**b**) Optimal number of clusters.

Figure 14a evaluates the scalability of the algorithms when using a 500-node network, while Figure 14b reveals the scalability of the algorithms in terms of the optimal number of clusters as analytically derived above: an algorithm using less clusters is more scalable than one that requires many clusters. The experimental results are shown in Figure 14b using a number of sensor nodes ranging from 100–500, a network of length M = 100 and $\epsilon_{fs}=10$ pJ/bit/m${}^{2}$. It is observed that the optimal number of clusters increases with the increase in the number of sensor nodes for all of the protocols. Compared to the DECSA and the MOCRN protocols, the SAC protocol revealed the least number of optimal clusters for the different number of sensor nodes considered. It is therefore more scalable than the two algorithms.

### 3.5. Traffic Engineering Performance

Objective: We conducted another set of experiments to evaluate the traffic engineering performance of the proposed SAC algorithm by looking at the energy frugality resulting from the distributed signaling process and the routing efficiency of the centralized clustering process. The energy frugality of our distributed signaling model using the LIBP-based signaling was compared to those of the RPL- and CTP-based signaling in terms of the average energy consumption and the radio duty cycle. The routing efficiency resulting from the centralized clustering process was measured by looking at the end-to-end packet delays and the packet delivery ratio resulting from running the different algorithms.

Network: The energy consumption was measured using different network sizes ranging from 10–100 nodes, while the radio duty cycle was considered for a 100-node network.

**Figure 15:**
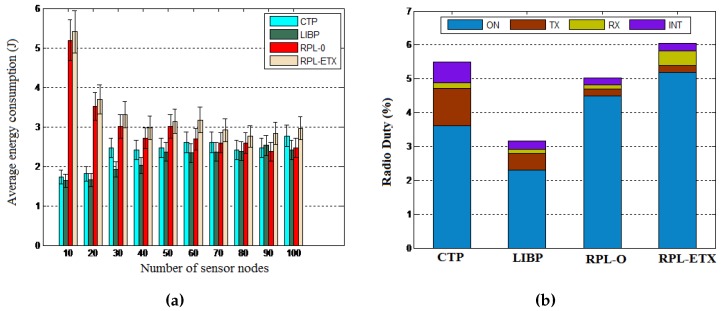
Signaling frugality. (**a**) Energy consumption; (**b**) Radio duty cycle.

**Figure 16:**
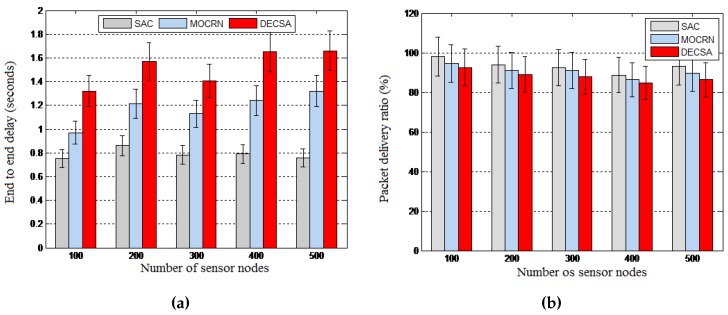
Routing efficiency. (**a**) End-to-end delay; (**b**) Packet delivery ratio.

Results: The results depicted by Figure 15 reveal the energy frugality of the LIBP-based signaling model compared to CTP and RPL in terms of energy consumption and radio duty cycle. The routing performance revealed by Figure 16 also showed that SAC outperforms DECSA and MOCRN in terms of end-to-end packet delay and the packet delivery ratio.

## 4. Conclusions and Future Works

A hybrid network is a network that integrates conventional wireless sensor nodes, sensor tags, hybrid RFID and a base station into the same network in order to provide different services to users in IoT environments. In such settings, sensors are designed to offer different services and, therefore, have different energy patterns, which may lead to network energy imbalance if not managed carefully. This paper proposed the SAC routing protocol to improve energy management in a hybrid network designed to identify objects and sense their environments by using hybrid sensor/RFID devices. The proposed routing protocol uses a centralized mechanism to address the energy imbalance problem by letting the base station select cluster heads depending on the residual energy of nodes, energy properties, the node’s type and service to be delivered and the position of nodes in the network. Experiments were conducted in a hybrid emulated/simulated IoT environment to compare the efficiency of the proposed SAC algorithm to the LEACH and LEACH-C protocols and the more recent DECSA and MOCRN protocols. It was observed, from these experiments, that the SAC protocol achieved the best energy management and better traffic engineering performance, leading to the longest life span.

Following the model presented in [21], experiments were conducted in parallel with those proposed in this paper to assess the relevance of using embedded boards, such as the raspberry pi, as smart gateways for the centralized clustering process proposed in this paper, as these devices are energy frugal and can be deployed pervasively and unattended with solar harvested energy in different IoT applications. Their efficient use in long-distance deployments [22] is another issue that is currently being addressed using different radio propagation models in different frequency bands, both ISM frequencies and white spaces. The experimental results related to this work are expected to be published as future work. Multipath routing [23,24] is often listed besides clustering as another routing approach for providing quality of service (QoS) in sensor networks. A comparison between the clustering model proposed in this paper and multipath routing is another avenue for future research work.

## References

[1] Mitrokotsa A., Douligeris C. (2009). Integrated RFID and Sensor Networks: Architectures and Applications.

[2] Eubanks W.W. (2008). Payment Card Interchange Fees: An Economic Assessment.

[3] Niu X., Huang X., Zhao Z., Zhang Y., Huang C., Cui L. The Design and Evaluation of a Wireless Sensor Network for Mine Safety Monitoring. Proceedings of the IEEE Global Telecommunications Conference (GLOBECOM’07).

[4] Pei Z., Deng Z.A. A Distributed Location Algorithm for Underground Miners Based on Rescue Robot and Coal-Mining Wireless Sensor Networks. Proceedings of the IEEE Conference on Robotics, Automation and Mechatronics.

[5] Hospital Using RTLS to Monitor Patients’ Conditions. http://www.rfidjournal.com/articles/view?6685.

[6] Jurdak R., Ruzzelli A.G., Hare G. Multi-Hop RFID Wake-Up Radio: Design, Evaluation and Energy Tradeoffs. Proceedings of the 17th International Conference on Computer Communications and Networks (ICCCN).

[7] Martin J. (2014). Hybrid RFID Sensors: Design, Implementation and Application. Master Thesis.

[8] Karbab E., Djenouri D., Boulkaboul S., Bagula A. Car Park Management with Networked Wireless Sensors and Active RFID. Proceedings of the IEEE International Conference on Electro/Information Technology (EIT).

[9] Bagula A., Castelli L., Zennaro M. (2015). On the Design of Smart Parking Networks in the Smart Cities: An Optimal Sensor Placement Model. Sensors.

[10] Heinzelman W.R., Chandrakasan A., Balakrishnan H. Energy Efficient Communication Protocol for Wireless Sensor Networks. Proceedings of the 33rd Annual Hawaii International Conference on System Sciences.

[11] Heinzelman W.B., Chandrakasan A.P., Balakrishnan H. (2002). An Application-Specific Protocol Architecture for Wireless Microsensor Networks. IEEE Trans. Wireless Commun..

[12] Yong Z., Pei Q. (2012). A energy-efficient clustering routing algorithm based on distance and residual energy for wireless sensor networks. Procedia Eng..

[13] Nam C.S., Bae S.T., Chung J.W., Shin D.R. (2013). Multihop-Based Optimal Cluster Heads Numbers Considering Relay Node in Transmission Range of Sensor Nodes in Wireless Sensor Networks. Int. J. Distrib. Sens. Netw..

[14] Antoine B., Djenouri D., Karbab E. (2013). On the Relevance of Using Interference and Service Differentiation Routing in the Internet-of-Things. Lect. Notes Comput. Sci..

[15] Kulik J., Heinzelman W., Balakrishnan H. (2002). Negotiation-based protocols for disseminating information in wireless microsensor networks. Wirel. Netw..

[16] Kim S., Lee S., An S. Reader Collision Avoidance Mechanism in Ubiquitous Sensor and RFID Networks. Proceeedings of the WiNTECH’06.

[17] Al-Karaki J.N., Kamal A.E. (2004). Routing techniques in wireless sensor networks: A survey. IEEE Trans. Wirel. Commun..

[18] Wang A., Heinzelman W., Chandrakasan A. Energy-Scalable Protocols for Battery-Operated Microsensor Networks. Proceedings of the IEEE Workshop Signal Processing Systems (SiPS’99).

[19] Bagula A., Djenouri D., Karbab E. Ubiquitous Sensor Network Management: The Least Interference Beaconing Model. Proceedings of the IEEE PIMRC-2013 Conference.

[20] Ngqakaza L., Bagula A. Least Path Interference Beaconing Protocol (LIBP): A Frugal Routing Protocol for the Internet-of-Things. Proceedings of the IFIP Wired/Wireless Internet Communications WWIC 2014.

[21] Zennaro M., Bagula A.B. (2010). Design of a flexible and robust gateway to collect sensor data in intermittent power environments. Int. J. Sensor Netw..

[22] Zennaro M., Bagula A., Gascon D., Noveleta A.B. (2010). Planning and Deploying Long Distance Wireless Sensor Networks: The Integration of Simulation and Experimentation. Lect. Notes Comput. Sci..

[23] Bagula A.B. (2010). Modelling and Implementation of QoS in Wireless Sensor Networks: A Multi-constrained Traffic Engineering Model. Eur. J. Wirel. Commun. Netw..

[24] Bagula A.B., Krzesinski A.E. Traffic Engineering Label Switched Paths in IP Networks Using a Pre-Planned Flow Optimization Model. Proceedings of the 9th International Symposium on Modelling, Analysis, and Simulation of Computer and Telecommunication Systems.

